# Circular RNAs in Lung Cancer: Recent Advances and Future Perspectives

**DOI:** 10.3389/fonc.2021.664290

**Published:** 2021-07-06

**Authors:** Huan-Huan Chen, Tie-Ning Zhang, Qi-Jun Wu, Xin-Mei Huang, Yu-Hong Zhao

**Affiliations:** ^1^ Department of Clinical Epidemiology, Shengjing Hospital of China Medical University, Shenyang, China; ^2^ Clinical Research Center, Shengjing Hospital of China Medical University, Shenyang, China; ^3^ Department of Oncology, Shengjing Hospital of China Medical University, Shenyang, China; ^4^ Department of Pediatric, Shengjing Hospital of China Medical University, Shenyang, China; ^5^ Department of Endocrinology, Shanghai Fifth People’s Hospital, Fudan University, Shanghai, China

**Keywords:** circular RNA, lung cancer, biomarker, mechanisms, drug-resistance, radioresistance

## Abstract

Globally, lung cancer is the most commonly diagnosed cancer and carries with it the greatest mortality rate, with 5-year survival rates varying from 4–17% depending on stage and geographical differences. For decades, researchers have studied disease mechanisms, occurrence rates and disease development, however, the mechanisms underlying disease progression are not yet fully elucidated, thus an increased understanding of disease pathogenesis is key to developing new strategies towards specific disease diagnoses and targeted treatments. Circular RNAs (circRNAs) are a class of non-coding RNA widely expressed in eukaryotic cells, and participate in various biological processes implicated in human disease. Recent studies have indicated that circRNAs both positively and negatively regulate lung cancer cell proliferation, migration, invasion and apoptosis. Additionally, circRNAs could be promising biomarkers and targets for lung cancer therapies. This review systematically highlights recent advances in circRNA regulatory roles in lung cancer, and sheds light on their use as potential biomarkers and treatment targets for this disease.

## Introduction

Worldwide, lung cancer is the most commonly diagnosed cancer with the highest mortality (1). Every year, approximately 1.82 million people are diagnosed, and 1.6 million die from the disease (2). The 5-year survival rate varies from 4–17% depending on stage and geographical differences (3). Approximately 85% of lung cancers are non-small cell lung cancers (NSCLCs), mainly including lung adenocarcinoma (LUAD) and lung squamous cell carcinoma (LUSC) (4, 5). For decades, researchers have studied disease mechanisms, occurrence rates and disease development, however, the mechanisms underlying disease progression are not yet fully elucidated, thus an increased understanding of disease pathogenesis is key to developing new strategies towards specific disease diagnoses and targeted treatments. In recent years, increased evidence has suggested that abnormal circular RNA (circRNA) expression is associated with several diseases, e.g., cardiovascular disease (6), osteoarthritis (7) and different cancers, including prostate (8), glioma (9) and breast cancer (10), etc However, the precise functions of circRNAs in lung cancer remain unclear.

CircRNAs were first discovered in the 1970s, and are a class of non-coding RNAs widely expressed in eukaryotic cells (11–15). Originally, they were believed to be rare products arising from mRNA splicing errors (15), however the first observations using electron microscopy in 1979 suggested these RNAs existed as circular forms (16). In the 21^st^ century, ubiquitous circRNA expression detection has been greatly facilitated by RNA sequencing (RNA-seq) technologies and bioinformatics, which have allowed researchers to investigate millions of short sequencing reads representing all RNA isoforms. CircRNAs are believed to be differentially expressed, and appear to play opposing roles in lung cancer etiology. For instance, circFGFR1 (17) is upregulated in NSCLC and promotes lung cancer progression, while circPTK2 (18) and circNOL10 (19) are downregulated and inhibit lung cancer development. Hence, this review comprehensively encompasses the latest contemporary research, and focuses on the emerging, divergent roles of circRNAs in lung cancer. A basic overview of circRNAs, their role in lung cancer, and the limitations of current research are explored.

## CircRNA Overview

**Figure 1:**
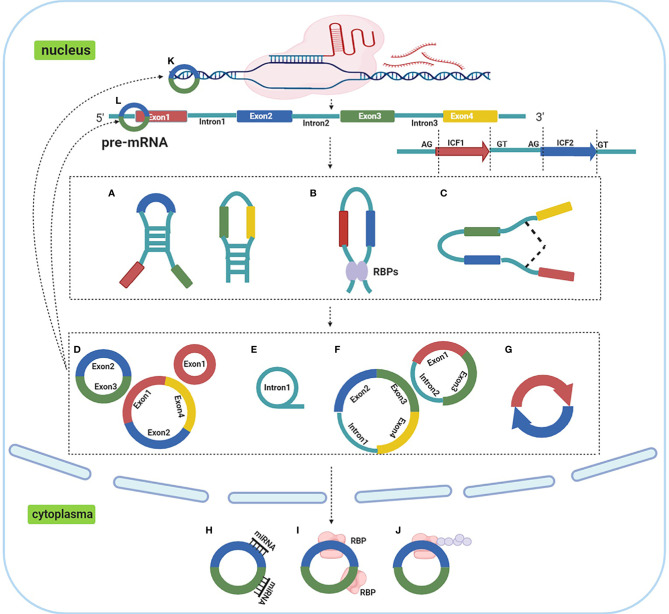
Biogenesis, categories and functions of circRNAs. **(A)**. reverse complementary intronic sequence paring; **(B)** RNA binding protein regulation; **(C)** alternative splicing; **(D)**. ecircRNAs; **(E)** ciRNAs; **(F)** ElciRNAs; **(G)** intergenic circRNAs; **(H)**. act as ceRNA or miRNA molecular sponges; **(I)** interacting with RNA binding proteins; **(J)** peptide translation; **(K)** transcriptional regulation; **(L)** modulating miRNA stability.

CircRNAs are covalently-closed molecules lacking 5’ to 3’ ends, polarity and a polyadenylated tail, with single-stranded transcripts derived from pre-mRNA (20). These characteristics provide them a higher tolerance to RNA exonucleases. The molecular structure is highly stable, they are highly abundant and incredibly diverse, they exhibit tissue specific expression patterns, and they are known to be involved in cellular differentiation and pluripotency (21–23). The majority of reported circRNAs are non-coding, while some have been reported to encode polypeptides or proteins (24). CircRNAs are divided into four categories, based on their origin (**Figure 1**) (25); 1) circRNAs derived from exons, exonic circRNAs (ecircRNAs), 2) circular intronic RNAs (ciRNAs) derived from introns, 3) exon-intron circRNAs (ElciRNAs) derived from a combination of exons and introns, and 4) intergenic circRNAs (26). Several hypotheses have been proposed for circRNA biogenesis (27), amongst which alternative splicing, reverse complementary intronic sequence paring, or RNA binding protein regulation are the most accepted.

Emerging evidence has suggested that circRNAs participate in various cellular biological processes including cell proliferation, differentiation, metastasis, senescence, apoptosis, etc (28), and are associated with lung cancer occurrence and development (29). CircRNAs functions primarily through the following five mechanisms; 1) act as competing endogenous RNA (ceRNA) or microRNA (miRNA) molecular sponges, 2) transcriptional regulation, 3) interacting with RNA binding proteins, 4) modulating miRNA stability and, 5) peptide translation (25, 30). Of these mechanisms, it is accepted that circRNAs act as miRNA sponges to regulate gene expression, and similarly, may have diagnostic or therapeutic potential for lung cancer treatment (31).

## The Role of CircRNAs in Lung Cancer

### CircRNAs Regulate Lung Cancer Cell Proliferation, Migration and Invasion

#### Cancer Associated Signaling Pathways

##### The Wnt/β-Catenin Pathway

Wnt activation has been observed in colorectal (32), breast (33) and lung cancer (34) and contributes to tumor onset and progression (35). CircRNAs are believed to promote or inhibit lung cancer cell proliferation, migration and invasion by regulating activation of the Wnt/β-catenin dependent or canonical pathway. CircRNAs regulating this pathway are summarized (**Table 1**).

**Table 1:** The circRNAs regulate lung cancer proliferation, migration and invasion via Wnt/β-catenin pathway.

NO.	Year	circRNA	Level	miRNA	Target Gene	Key Points of Investigation	Ref
1	2020	circ_0007142	up	miR-186	FOXK1	Promote LC progression by activating Wnt/β-catenin pathway via miR-186/FOXK1 axis	(36)
2	2018	circ_001569	up	–	–	Promote LC progression by activating Wnt/β-catenin pathway	(37)
3	2019	hsa_circ_000984	up	–	–	Promote LC progression by activating Wnt/β-catenin pathway	(38)
4	2020	circRNA FOXP1	up	miR-185-5p	Wnt1	Promote LC progression by activating Wnt1 signaling via sponging miR-185-5p	(39)
5	2020	circ-SOX4	up	miR-1270	PLAGL2	Promote LC progression by activating Wnt/β-catenin pathway via miR-1270/PLAGL2 axis	(40)
	2020			–	c-MYC	Promote LC progression by activating Wnt/β-catenin pathway	(41)
6	2019	circ_0001946	up	miR-135a-5p	SIRT1	Promote LC progression by activating Wnt/β-catenin pathway via miR-135a-5p/SIRT1 axis	(42)
7	2016	circ-ITCH	down	miR-7/miR-214	ITCH	Inhibit LC progression by inactivating Wnt/β-catenin pathway via miR-7 and miR-214/ITCH axis	(43)
8	2017	hsa_circ_0043256	–	miR-1252	ITCH	Inhibit LC progression by inactivating Wnt/β-catenin pathway via miR-1252/ ITCH axis	(44)
9	2019	hsa_circ_0007059	down	miR-378	–	Inhibit LC progression by inactivating Wnt/β-catenin and ERK1/2 pathway	(45)
10	2020	circ-IGF1R	down	miR-1270	VANGL2	Inhibit LC progression by inactivating Wnt/β-catenin pathway via miR-1270/VANGL2	(46)
11	2019	circ_0006427	down	miR-6783-3p	DKK1	Inhibit LC progression by inactivating Wnt/β-catenin pathway via miR-6783-3p/DKK1 axis	(47)

LC, lung cancer; SIRT1, sirtuin 1; ITCH, Itchy E3 ubiquitin protein ligase.

CircRNAs overexpressed in lung cancer cells and tissue drive *in vitro* cancer progression. For example, Liu et al. indicated that the circ_0007142/miR-186/FOXK1 axis played an important role in LUAD cell progression by activating the Wnt/β-catenin signaling pathway (36). Ding et al. observed that circ_001569 promoted cell proliferation by regulating the Wnt/β-catenin pathway in LUAD cells, whereas elevated circ_001569 expression demonstrated a poorer survival outcome (37). Li et al. also observed that hsa_circ_000984 exerted oncogenic functions by modulating Wnt/β-catenin pathway activation in LUAD cells (38). In other research, circRNA FOXP1 promoted LUAD cell proliferation by regulating the miR-185-5p/Wnt1 signaling pathway (39).

In addition, the role of several circRNAs with oncogenic functions in lung cancer mediated by the Wnt canonical pathway, were also demonstrated in *in vivo* studies. For instance, circ-SOX4 had oncogenic roles through the miR-1270/PLAGL2 axis, and subsequently activated the WNT signaling pathway in LUAD cells (40). *In vivo* data from this study showed that tumor volume and weight of xenograft nude mice were smaller in the circ-SOX4 silenced LUAD cell group when compared with the negative control (NC) group. Another study revealed that circ-SOX4 interacted with c-MYC by activating the Wnt/β-catenin pathway in LUAD cells, with circ-SOX4 down-regulating suppressed lung tumor-initiating cell proliferation, self-renewal, migration and invasion (41). Moreover, Yao et al. observed that circ_0001946 promoted LUAD cell growth by sponging miR-135a-5p to upregulate sirtuin 1 (SIRT1) expression (42). Moreover, *in vivo* experiments demonstrated that knockdown of circ_0001946 markedly suppressed tumor growth in nude mice. SIRT1 is a positive regulator of the Wnt/β-catenin signaling pathway (48, 49).

Several circRNAs have been downregulated in NSCLC, and appear to play an opposing role in lung cancer progression *via* inactivation of the Wnt canonical pathway. Itchy E3 ubiquitin protein ligase (ITCH) is a vital negative regulator of canonical Wnt signaling (44). Wan et al. found that *circ-ITCH* acted as sponge for oncogenic miR-7 and miR-214 to enhance ITCH expression, and thus suppressed activation of Wnt/*β*-catenin signaling (43). Tian et al. observed that hsa_circ_0043256 was upregulated in cinnamaldehyde treated NSCLC cells, and inhibited LUAD cell proliferation and induced apoptosis through ITCH in LUAD cell lines (44). Mechanistically, hsa_circ_0043256 functions as an miR-1252 sponge to directly target ITCH and inhibit Wnt/*β*-catenin pathway (44). Additionally, overexpressed hsa_circ_0007059 inhibited epithelial-to-mesenchymal transition (EMT) expression of the EMT proteins; vimentin, twist1 and zeb1, and elevated E-cadherin expression by suppressing miR-378 (45). Western blot assays revealed that overexpressed hsa_circ_0007059 inhibited Wnt3a, β-catenin and p-ERK1/2 expression in LUAD cell lines. Xu et al. observed that circ-IGF1R overexpression inhibited LUAD migration and invasion *via* the miR-1270/VANGL2 axis (46). *VANGL2* is a Wnt signaling pathway related gene, and circ-IGF1R overexpression downregulated the Wnt pathway associated proteins, β‐catenin1 and vimentin (46). Yao et al. indicated that circ_0006427 overexpression effectively suppressed LUAD cell proliferation, migration and invasion (47) by inactivating the Wnt/β-catenin signaling pathway *via* miR-6783-3p sponging and DKK1 upregulation.

Based on these *in vitro* and *in vivo* studies, the Wnt pathway appears to be an important signaling pathway for circRNAs in regulating lung cancer progression, thus further exploration as a clinical therapy target is warranted. While these mechanisms are primarily *via* the canonical Wnt pathway, circRNA effects towards non-canonical pathways (Wnt/Ca^2+^ pathway and planar cell polarity pathway) are rarely studied, therefore these pathways could potentially open up new exploratory avenues for lung cancer research.

##### The Mitogen-Activated Protein Kinase (MAPK) Signaling Pathway

MAPK cascades are key signaling pathways which regulate a variety of cellular processes (e.g. proliferation, differentiation and apoptosis) (50, 51), and include four signaling families: the MAPK/extracellular signal-regulated kinase (ERK) family or the classical pathway, Big MAP kinase-1 (BMK-1), c-Jun N-terminal kinase (JNK) and p38 signaling families (52). Several circRNAs are upregulated in lung cancer and participate in tumorigenesis *via* MAPK signaling pathways. Chen et al. observed that hsa_circ_0007580 sponged miR-545-3p and subsequently inhibited protein kinase Ca (PKCA) to promote NSCLC cell proliferation and invasion by activating p38/MAPK signaling (53). In a xenograft tumor model, downregulated hsa_circ_0007580 inhibited NSCLC tumorigenesis by inactivating p38/MAPK signaling. Moreover, circ_0074027 exerted oncogenic properties in NSCLC cells *via* sponging miR‐185‐3p to upregulate bromodomain‐containing protein 4 (BRD4) and MAPK‐activating death domain containing protein (MADD) expression levels (54). Zhang et al. found that hsa_circRNA_101237 promoted MAPK1 expression *via* miRNA-490-3p sponging, thereby affecting NSCLC proliferation, migration and invasion, *via* its role as an important onco-circRNA (55).

Interestingly, Wang et al. found that circ-ZKSCAN1 (hsa_circ_0001727) acted as a sponge for carcinogenic miR-330-5p to increase the expression of family with sequence similarity 83(FAM83)-member A, inhibit the MAPK signal transduction pathway, and promote NSCLC progress (56). Overexpression of circZKSCAN1 significantly decreased JNK, p38 and ERK expression in NSCLC cells (56). Accordingly, these studies suggested that circRNAs promoted lung cancer progression by activating MAPK signaling, except for circ-ZKSCAN1, which exerted its oncogenic role by inhibiting MAPK signal transduction.

##### The Nuclear Factor-κB (NF-κB) Signaling Pathway

Early studies revealed that the NF- κB signaling pathway played important roles in cancer proliferation and apoptosis (57–59). Evidence showed that circRNAs appeared to interact with the NF- κB signaling pathway during lung cancer progression. Liu et al. observed that circ_cMras, alpha-beta hydrolase domain 5 (ABHD5) and adipose triglyceride lipase (ATGL) were downregulated in LUAD tissue and cells (60). Furthermore, the upregulation of circ_cMras inhibited LUAD cell proliferation, migration and invasion, and inhibited *in vivo* tumor growth *via* the ABHD5/ATGL axis, which targeted the NF-κB signaling pathway.

#### Proteins Related to DNA Repair and RNA Splicing or Translation

##### High Mobility Group (HMG) Proteins

The HMG of proteins, i.e., HMGA, HMGB and HMGN are non-histone nuclear proteins, which modulate DNA repair efficiency in all the major cellular pathways, i.e., nucleotide excision, base excision, double-stand break and mismatch repair (61), but also participate in cancer onset and progression (62–64). Studies have indicated that circRNAs upregulated in NSCLC promote lung cancer progression by positively influencing HMGA2 and HMGB3 expression. For example, Xu et al. identified that aspartate beta-hydroxylase (ASPH) RNA (circASPH/hsa_circ_0084606) was regulated by HMGA2 overexpression, by screening 6576 circRNAs using RNA-seq analysis (65). CircASPH promoted tumor growth in LUAD cells by sponging miR-370 and abrogating miR-370-mediated inhibition of HMGA2 (65). Li et al. also demonstrated that another circRNA, circ_100565 promoted proliferation, migration and invasion of NSCLC by upregulating HMGA2 *via* miR-506-3p sponging (66). Moreover, circEPSTI1 aggravated *in vitro* NSCLC progression by elevating HMGB3 expression *via* miR-145 sponging (67). Similarly, circEPSTI1 silencing restrained NSCLC tumor growth *in vivo (* 67). Zhou et al. found that circRNA_102179 facilitated NSCLC proliferation, migration and invasion *via* the miR-330-5p/HMGB3 axis (68).

##### The Trinucleotide Repeat Containing 6 (TNRC6 or GW182) Family of Proteins

This family of proteins are core components of RNA interference (RNAi) molecules, and consist of three paralogs, i.e., TNRC6A, TNRC6B and TNRC6C (69). Argonaute and TNRC6 proteins, which form the RNA-induced silencing complex, mediate the fine-tuning of gene expression and are involved in several key biological processes (70), thereby greatly influencing disease development, especially cancer (71). The circRNAs, circABCC4 and circ0006916, were shown to interact with TNRC6 to positively and negatively regulate lung cancer progression, respectively. CircABCC4 was upregulated in LUAD and promoted cell proliferation, migration and invasion *via* miR-3186-3p sponging to upregulate TNRC6B (72). However, Circ0006910 was downregulated in lung cancer cells and tissue, and acted as a tumor suppressor in NSCLC *via* miR-522-3p sponging, and inhibiting pleckstrin homology domain and leucine rich repeat protein phosphatase 1 (PHLPP1) activity, thus inhibiting cell proliferation (73). Similarly, TNRC6A may also promote circ0006916 expression (73).

##### The MYC Family

The MYC oncogene family is dysregulated in > 50% of cancers, and is frequently associated with poor prognosis and unfavorable patient survival rates (74). The MYC oncogene encodes the transcription factor, MYC, which triggers selective gene expression amplification to promote cancer cell growth and proliferation (75). Two circRNAs were upregulated in lung cancer tissues and cell lines, with oncogenic roles that positively regulated MYC expression. Hsa_circRNA_103809 promoted lung cancer cell proliferation and invasion by promoting ZNF121 expression *via* miR-4302 sponging, and thus elevated ZNF121 expression levels, subsequently increasing MYC levels in lung cancer (76). Additionally, Zhang et al. indicated that circRNA_010763 promoted NSCLC proliferation, migration and invasion by sponging miR-715 to modulate its inhibitory effects on oncogenic c-MYC (77).

##### The Forkhead Box Protein M1 (FOXM1)

FOXM1 is a critical proliferation-associated transcription factor widely expressed during the cell cycle (78). In most cancers, including lung cancer, FOXM1 is oncogenic in nature thanks to its repeated upregulation, thereby generating a poor prognosis for patients (79, 80). Data from two studies showed that circRNAs upregulated in lung cancer tissue and cells, promoted lung cancer progression by regulating FOXM1 *via* miRNA sponging. Cheng et al. suggested that elevated circTP63 promoted LUSC cell proliferation both *in vivo* and *in vitro*, however, the ectopic expression of *circTP63* exerted no significant effects on LUSC migration and invasion (81). Mechanistically, *circTP63* overexpression promoted cell progression from the G1/S to the G2/M phase, suggesting increased cell cycle progression *via* sponging miR-873-3p to upregulate FOXM1. This subsequently regulated expression of the cell cycle related proteins, CENPA, histone H3 variant and CENPB heterochromatin (81). Additionally, Lu et al. observed that circHIPK3 overexpression promoted both cell proliferation and invasion *in vitro*, and tumorigenesis and metastasis *in vivo*, by sponging miR-149 and subsequently upregulating FOXM1 expression (82).

Moreover, a circRNA derived from FOXM1was closely associated with NSCLC progression. Yu et al. indicated that has_circ_0025039 was derived from exons 4 and 5 of FOXM1, labeling it circFOXM1 (83). This circRNA was upregulated in NSCLC tissue, potentially predicting an unfavorable overall survival (OS) for NSCLC patients (83). These findings suggested that circFOXM1 may have an oncogenic role in NSCLC progression by influencing the expression of cell cycle-related genes *via* miR-614 sponging, and FAM83D upregulation. Overall, these studies indicated that circRNAs promoting FOXM1 expression or derived from FOXM1 itself, could promote lung cancer progression, suggesting FOXM1 may function as a new molecular target for lung cancer treatment.

#### Cancer Associated Biological Processes

##### The EMT Process

EMT is a biological process where non-motile epithelial cell changes occur in a mesenchymal phenotype, but with invasive capacities (84). This phenomenon has been well documented in multiple biological processes, including embryogenesis, fibrosis, tumor progression and metastasis (84, 85). The EMT process plays a key role in the migration and invasion of malignant tumors, including NSCLC (86). Several circRNAs are upregulated in NSCLC, with oncogenic roles to positively regulate *in vitro* EMT processes. For instance, circ-LDLRAD3 promoted proliferation and EMT in NSCLC cells by downregulating miR-137, and subsequently upregulating glutamine transporter solute carrier family A1 member 5 (SLC1A5) (87). Notably, SLC1A5 was identified as participating in NSCLC progression and regulation, and similarly, its inactivation inhibited NSCLC cell viability (88). The circ_0012673 also facilitated LUAD proliferation and invasion (89). Loss-of-function studies indicated that circ_0012673 knockdown restricted proliferation, motility and EMT, but induced apoptosis by targeting miR-320a, and subsequently upregulating LIM domain kinase 1 in LUAD cell lines (89). Li et al. also noted that hsa_circ_0079530 upregulation promoted NSCLC cell migration and invasion by regulating EMT processes (90). Liu et al. found that hsa_circ_0023404 affected the expression of EMT related proteins, by regulating the miR-217/zinc finger E-box-binding homeobox 1 (ZEB1) axis, and contributing to *in vitro* NSCLC cell growth (91).

Moreover, several *in vivo* and *in vitro* studies have also indicated that circRNAs facilitate lung cancer progression by promoting EMT processes. Qu et al. observed that hsa_circ_0020123 enhanced proliferation, migration and invasion, while inhibiting *in vitro* NSCLC apoptosis, by suppressing miR-144 and upregulating ZEB1 and EZH2, respectively (92). Further studies also indicated that hsa_circ_ 0020123 knockdown inhibited NSCLC growth and metastasis both *in vitro* and *in vivo* (92). In their study, Chi et al. suggested that circPIP5K1A (Circ_0014130) potentially functioned as an miR-600 sponge to facilitate NSCLC proliferation and metastasis, by promoting hypoxia-inducible factor (HIF)-1α and reversing the inhibitory effects of miR-600 on EMT-related proteins (93). Furthermore, *in vivo* studies further illustrated that circPIP5K1A silencing suppressed tumor growth and pulmonary metastasis (93). Wang et al. found that circP4HB enhanced EMT processes in NSCLC *via* miR-133a-5p sequestration, leading to the *in vitro* upregulation of vimentin, and facilitating *in vivo* xenograft metastasis (94). Additionally, hsa_circ_0007534 positively regulated cell migration and invasion by affecting EMT in NSCLC cells, and promoting tumor growth in nude mice (95), however the underlying hsa_circ_0007534 mechanisms have yet to be elucidated.

However, several circRNAs are downregulated in NSCLC, and inhibit disease progression both *in vitro* and *in vivo* by negatively regulating EMT processes. Wang et al. found that hsa_circ_00008305 (circPTK2) acted as a sponge for miR-429/miR-200b-3p, and was positively correlated with transcriptional intermediary factor 1-γ (TIF1-γ) expression in human NSCLC tissue (18). CircPTK2 overexpression augmented TIF1-γ expression, and inhibited TGF-β-induced EMT and NSCLC cell invasion. *In vivo* studies showed that circPTK2 overexpression suppressed NSCLC cell metastasis (18). CircPTPRA also suppressed EMT processes in NSCLC cell lines and reduced *in vivo* metastasis in the murine xenograft model, by sequestering miR-96-5p and upregulating Ras association domain-containing protein 8 (RASSF8) (96). These findings have provided new EMT-mediated insights into the role of circRNAs in lung cancer.

##### Glycolysis

**Figure 2:**
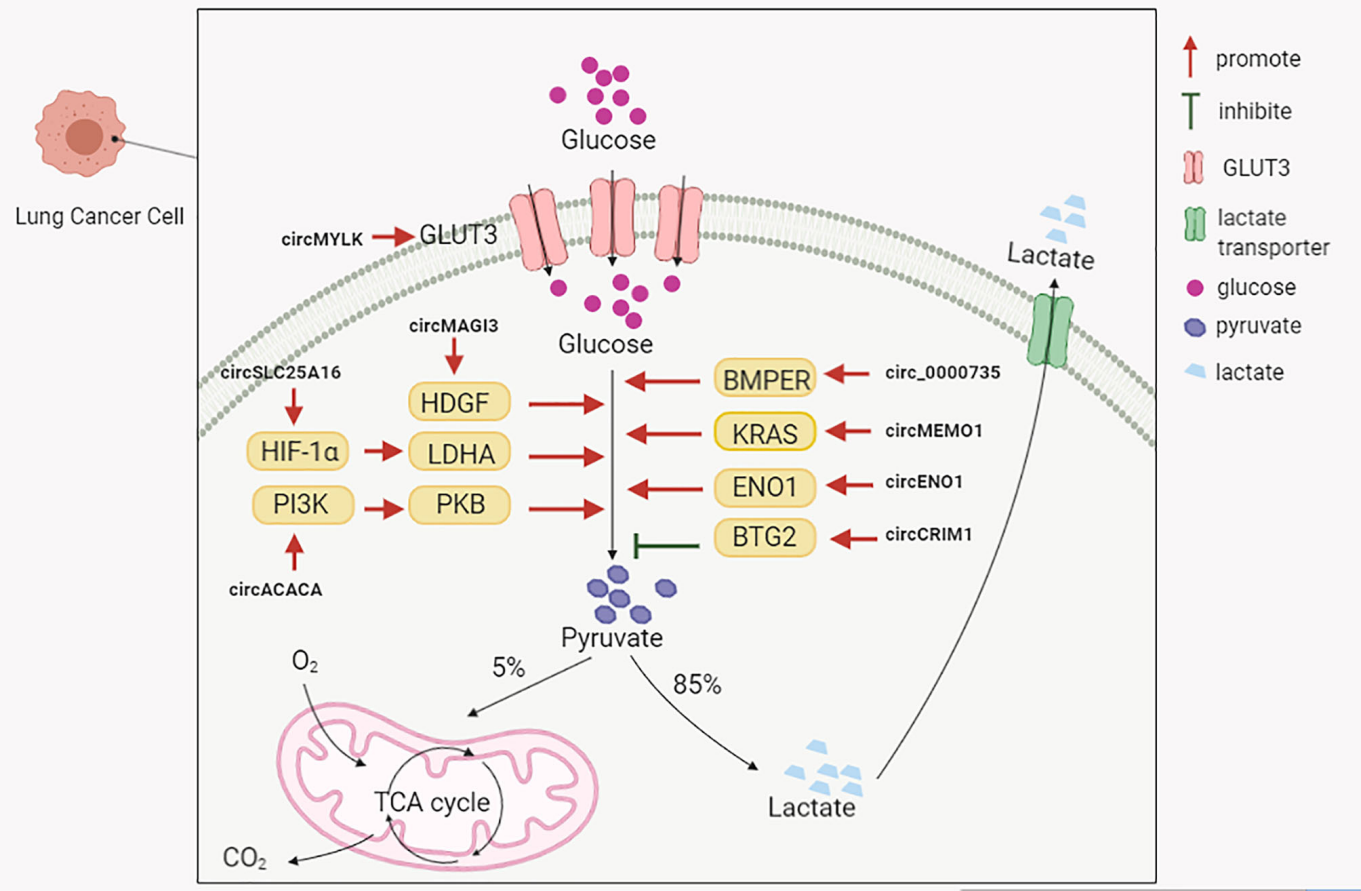
The abnormal expression of circRNAs in lung cancer influencing glycolytic mechanisms.

Glycolysis is the predominant energy producing pathway for cancer cells under both aerobic and hypoxic conditions, and is a biochemical fingerprint representing one of the “hallmarks of cancer” (97). The abnormal expression of circRNAs in lung cancer appears to influence glycolytic mechanisms (**Figure 2**). Several circRNAs upregulated in NSCLC could positively regulate glycolysis to promote disease progression. For instance, Guo et al. observed that circMAGI3 functioned as a sponge for miR-515-5p to promote its target gene, hepatoma-derived growth factor (HDGF) expression, thereby accelerating glycolytic mechanisms in NSCLC (98). Hong et al. found that circSLC25A16 accelerated glycolysis and promoted NSCLC proliferation by interacting with miR-488-3p and enhancing HIF-1α expression, which transcriptionally activated lactate dehydrogenase A (LDHA) (99). A study by Wu et al. indicated that circ-ACACA promoted *in vitro* NSCLC proliferation, migration and glycolysis by negatively regulating miR-1183 to activate the phosphoinositide 3-kinase/protein kinase B(PI3K/PKB) pathway (100). Huang et al. observed that circ_0000735 overexpression enhanced NSCLC proliferation, migration, invasion, and glycolysis by targeting the miR-940/bone morphogenetic protein binding endothelial cell precursor-derived regulator (BMPER, a secreted glycoprotein) axis (101). Ding et al. reported that circ-MEMO1 elevated expression in NSCLC patients was associated with a poor prognosis (102). Elevated circ-MEMO1 levels accelerated cell proliferation, cell cycle progression and aerobic glycolysis, and inhibited NSCLC apoptosis *via* the miR-101-3p/KRAS axis (102). Equally, the circRNA knockdown or silencing of these aforementioned molecules also suppressed tumor growth effects in *in vivo* xenograft assays.

Moreover, circRNA-ENO1 promoted glycolysis so as to promote LUAD proliferation, migration and EMT processes *via* the miR-22-3p/enolase 1 (ENO1) axis (103). Additionally, also circ-ENO1 promoted tumor growth and metastasis *in vivo*. Xiong et al. observed that circMYLK overexpression was closely associated with a poor prognosis in NSCLC patients (104). At the molecular level, circMYLK promoted glycolysis and NSCLC proliferation by sponging miR-195-5p, and up-regulating the expression of glucose transporter member 3 (GLUT3) (104).

However, distinct to the functionalities of these aforementioned circRNAs, Zhang et al. observed that circCRIM1 and BTG anti-proliferation factor 2 (BTG2) were downregulated, and miR-125b-5p upregulated in LUAD tissue and cells (105). CircCRIM1 upregulation inhibited *in vitro* LUAD cell migration, invasion, EMT processes and glycolysis by sponging miR-125b-5p to promote BTG2 expression, and negatively affect *in vitro* tumor growth (105). These studies demonstrated that circRNAs regulated glucose metabolism during lung cancer, and are potential therapeutic targets for the disease.

#### Other Mechanisms

In addition to the circRNA mechanisms summarized above, circRNAs may also function *via* other distinct mechanisms (**Table 2**).

**Table 2:** The circRNAs regulate lung cancer proliferation, migration and invasion via other mechanisms.

NO.	Year	circRNA	Level	miRNA	Target Gene	Key Points of Investigation	Ref
1	2020	circGFRA1	up	miR-188-3p	–	Promote LC progression by activating PI3K/AKT signaling pathway via sponging miR-188-3p	(106)
2	2019	circ-CAMK2A (hsa_circ_0128332)	up	miR-615-5p	fibronectin 1	Promote LC progression by increasing MMP2 and MMP9 expression via miR-615-5p/fibronectin 1 axis	(107)
3	2018	circMAN2B2	up	miR-1275	FOXK1	Promote LC progression via miR-1275/ FOXK1 axis	(108)
4	2020	circHIPK3	up	miR-107	BDNF	Promote LC progression via miR-107/ BDNF axis	(109)
5	2019	circFOXM1	up	miR-1304-5p	PPDPF and MACC1	Promote LC progression via miR-1304-5p/PPDPF/MACC1 axis	(110)
6	2020	circ_0000376	up	miR-1182	NOVA2	Promote LC progression via miR-1182/NOVA2 axis	(111)
7	2019	circ_0000735	up	miR-1179/-1182	–	Promote LC progression via sponging miR-1179/-1182	(112)
8	2019	circ_0043278	up	miR-520f	ROCK1,CDKN1B, and AKT3	Promote LC progression via sponging to increase ROCK1, CDKN1B, and AKT3 expression	(113)
9	2018	circFGFR3	up	miR-22-3p	Gal-1	Promote LC progression by activating AKT and ERK1/2 signaling pathways via smiR-22-3p/Gal-1 axis	(114)
10	2017	hsa_circ_0000064	up	–	–	Promote LC progression by increasing casepase-3,-9,Bax,MMP-2 and MMP-9 expression	(115)
11	2020	circ_0020123	up	miR-590-5p	THBS2	Promote LC progression via miR-590-5p /THBS2 axis	(116)
12	2019	circARHGAP10	up	miR-150-5p	GLUT-1	Promote LC progression via miR-150-5p/GLUT-1 axis	(117)
13	2020	circ-ABCB10(circRNA‐0008717)	up	–	KISS1	Promote LC progression by suppressing KISS1	(118)
	2018			miR‐1252	FOXR2	Promote LC progression via miR‐1252/ FOXR2 axis	(119)
	2020			miR-584-5p	E2F5	Promote LC progression via miR-584-5p/E2F5 axis	(120)
	2020			miR-217	–	Promote LC progression via sponging miR-217	(121)
14	2019	circ_0003645	up	miR1179	TMEM14A	Promote LC progression via sponging miR-1179/ TMEM14A axis	(122)
15	2018	circ_0016760	up	miR1287	GAGE1	Promote LC progression via miR1287/GAGE1 axis	(123)
16	2019	circ_0026134	up	miR-1256/-1287	TCTN1 and GAGE1	Promote LC progression via miR-1256 and -1287 / TCTN1 and GAGE1 axis	(124)
17	2019	circ-CMPK1 (hsa_circ_0012384)	up	miR-302e	cyclin D1	Promote LC progression via miR-302e/ cyclin D1 axis	(125)
18	2019	circZFR	up	miR-101-3p	CUL4B	Promote LC progression via miR-101-3p /CUL4B axis	(126)
19	2018	hsa_circ_0012673	up	miR-22	ErbB3	Promote LC progression via miR-22/ErbB3	(127)
20	2019	circ-RAD23B	up	miR-593-3p/-653-5p	CCND2 and TIAM1	Promote LC progression via miR-593-3p/CCND2 and miR-653-5p/TIAM1 axis	(128)
21	2020	circTIMELESS (hsa_circ_0000408)	up	miR‐136‐5p	ROCK1	Promote LC progression via miR‐136‐5p/ ROCK1 axis	(129)
22	2020	has_circ_ 0000326	up	miR-338-3p	RAB14	Promote LC progression via miR-338-3p/RAB14 axis	(130)
23	2020	hsa_circ_0087862	up	miR-1253	RAB3D	Promote LC progression via miR-1253/RAB3D axis	(131)
24	2018	circ-BANP	up	miR-503	LARP1	Promote LC progression via miR-503/LARP1	(132)
25	2020	circ_0016760	up	miR-577	ZBTB7A	Promote LC progression via miR-577/ZBTB7A axis	(133)
26	2020	circ-ZNF609	up	miR-1224-3p	EVT1	Promote LC progression via miR-1224-3p/ETV1 axis	(134)
27	2020	circ_POLA2	up	miR-326	GNB1	Promote LC progression via miR-326/ GNB1 axis	(135)
28	2018	hsa_circ_0003998	up	miR-326	Notch1	Promote LC progression via miR-326/Notch1 axis	(136)
29	2020	circMET	up	miR-145-5p	CXCL3	Promote LC progression via miR-145-5p/CXCL3	(137)
30	2020	circBIRC6	up	miR-145	FSCN1 and S6K1	Promote LC progression via miR-145/ FSCN1 and S6K1 axis	(138)
31	2020	circRNA_103993	up	miR-1271	ERG	Promote LC progression via miR-1271/ERG axis	(139)
32	2020	hsa_circ_0001588	up	miR-524-3p	NACC1	Promote LC progression via miR-524-3p /NACC1	(140)
33	2020	circDLGAP4	up	miR-143	CDK1	Promote LC progression via miR-143/ CDK1 axis	(141)
34	2020	circRNA_001010	up	miR-5112	CDK4	Promote LC progression via miR-5112/CDK4 axis	(142)
35	2018	circPRKCI	up	miR-545/-589	E2F7	Promote LC progression via miR-545 and -589/E2F7 axis	(143)
36	2018	F-circEA-2a	–	–	–	Promote LC progression	(144)
37	2019	F-circSR (1and 2)	–	miR-150-5p/-194-3p/-515-5p	–	Promote LC progression	(145)
38	2019	circATXN7	up	–	–	Promote LC progression	(146)
39	2018	circFADS2 (hsa_circRNA_100833)	up	miR-498	–	Promote LC progression via sponging miR-498	(147)
40	2018	hsa_circ_0007385	up	miR-181	–	Promote LC progression via sponging miR-181	(148)
41	2020	circ_0014130	up	miR-142-5p	IGF-1	Promote LC progression via miR-142-5p/ IGF-1 axis	(149)
42	2020	circ_0000284	up	miR-377-3p	PD-L1	Promote LC progression via miR-377-3p/PD-L1 axis	(150)
43	2019	hsa_circ_0000211	up	miR-622	HIF1-α	Promote LC progression via miR-622/ HIF1-α axis	(151)
44	2020	circRNA CDR1as	up	miR-219a-5p	SOX5	Promote LC progression via miR-219a-5p/SOX5 axis	(152)
45	2018	hsa_circ_100395	down	miR-1228	TCF21	Inhibit LC progression via miR-1228/TCF21 axis	(153)
46	2019	circCRIM1	down	miR‐93/‐182	LIFR	Inhibit LC progression via miR‐93 and miR‐182/LIFR axis	(154)
47	2018	circ_0001649	down	miR-331-3p/-338-5p	–	Inhibit LC progression via sponging miR-331-3p/-338-5p	(155)
48	2020	circ_0072309	down	miR-580-3p	–	Inhibit LC progression via sponging miR-580-3p	(156)
49	2018	hsa_circ_0046264	down	hsa-miR-1245	BRCA2	Inhibit LC progression via has_miR_1245/BRCA2 axis	(157)
50	2020	circ-LARP4	down	–	SMAD7	Inhibit LC progression via upregulating SMAD7	(158)
51	2020			miR-21-5p		Inhibit LC progression via sponging miR-21-5p	(159)
52	2020	hsa_circ_11780	down	miR-544a	FBXW7	Inhibit LC progression via miR-544a/ FBXW7 axis	(160)
53	2020	circ_EPB41L2	down	miR-211-5p	CDH4	Inhibit LC progression via miR-211-5p/ CDH4 axis	(161)
54	2019	Circ-MTO1 (hsa_circ_0007874)	down	miR-17	QKI-5	Inhibit LC progression by inactivating of Notch signaling pathway via miR-17/QKI-5 axis	(162)
55	2020	circ-SLC7A6	down	miR-21	–	Inhibit LC progression via sponging miR-21	(163)

BDNF, brain-derived neurotrophic factor; PPDPF, pancreatic progenitor cell differentiation and proliferation factor; MACC1, metastasis-associated in colon cancer 1; Gal-1, galectin-1; THBS2, Thrombospondin 2; GLUT-1, glucose transporter-1; KISS1, kisspeptin-1; FOXR2, Forkhead box 2; TMEM14A, transmembrane protein 14A; GAGE1, G antigen 1; TCTN1, Tectonic1; CUL4B, cullin 4B; ErbB3, erb-b2 receptor tyrosine kinase 3; ROCK1, coiled‐coil containing protein kinase 1; ZBTB7A, Zinc finger and BTB domain containing 7A; GNB1, G protein subunit beta 1; NACC1, nucleus accumbens-associated protein 1; CDK1, cyclin-dependent kinase 1; BRCA2, breast-cancer susceptibility gene 2; FBXW7, F-Box and WD repeat domain containing 7; CDH4, Cadherin-4; IGF-1, insulin-like growth factor -1; TCF21, transcription factor 21.

Several circRNAs are upregulated in NSCLC, and exert oncogenic roles during lung cancer (108–152). For instance, the phosphatidylinositol 3-kinase-regulated protein kinase, Akt, plays an important role in cancer initiation and progression (164). Mammalian cells express three Akt isoforms (Akt1–3), which are encoded by three distinct genes (164). Yao et al. reported that circGFRA1 acted as an miR-188-3p sponge, to regulate NSCLC proliferation *via* the PI3K/Akt signaling pathway (106). Moreover, matrix metalloproteinases (MMPs) play vital roles in many biological processes (165). In particular, MMP-2 and MMP-9 are both implicated in NSCLC invasion and tumor metastasis (166). A recent study indicated that circ-CAMK2A (hsa_circ_0128332) up-regulated the expression levels of fibronectin 1 by sponging miR-615-5p, thereby increasing MMP-2 and MMP-9 expression levels to promote LUAD metastasis (107).

In contrast, some circRNAs are downregulated in NSCLC and have roles as cancer suppressors (154–163). For example, Chen *et al.* observed that hsa_circ_100395 overexpression dramatically inhibited *in vitro* NSCLC cell proliferation, arrested cell-cycle progression and reduced cell migration and invasion (153). Data from a flow cytometry (FACS) study indicated that hsa_circ_100395 overexpression increased cells arrested in G0/G1 phase, with fewer cells in S and G2/M phase (153). Mechanistically, hsa_circ_100395 promoted expression of transcription factor 21, also known as a suppressor, by sponging miR-1228 in lung cancer (153). These data exemplified the multi-mechanistic roles of circRNAs in lung cancer progression.

### CircRNAs Regulate Lung Cancer Cell Death

#### Signal Transducer and Activator of Transcription (STAT) Proteins

These proteins are cytoplasmic transcription factors implicated in many cellular biological processes (167). Numerous studies have reported that STAT overexpression enhances carcinogenesis and affects prognosis in cancer patients (168), including NSCLC (169, 170). Chen et al. investigated circHIPK3 mechanisms in regulating lung cancer cell death (171). Their data indicated that autophagy was induced upon loss of circHIPK3, *via* the miR124-3p-STAT3-PRKAA/AMPKa axis in STK11 mutant lung cancer cell lines (171). At the molecular level, circHIPK3 negatively regulated miR124-3p expression and subsequently upregulated STAT3 expression (171). Further research has indicated that STAT3 silencing and inhibition induced autophagy *via* the PRKAA/AMPKa pathway in STK11 mutant cells. A recent study provided evidence that silencing circHIPK3 induced lung cancer cell death and apoptosis, by sponging miR-124 and regulating miR-124 targets, including SphK1, STAT3 and CDK4 proteins (172). Additionally, Wang et al. showed that STAT3 activated circCCDC66 transcription, and thereby promoted circCCDC66 expression in NSCLC cells, and similarly circCCDC66 inhibited cell apoptosis *via* the miR-33a-5p/KPNA4 axis in these cells (173). These studies highlighted the key mechanistic interactions between circRNAs and STAT3 in regulating lung cancer death.

#### B-Cell Lymphoma 2 (Bcl-2) and/or Bcl-2-Associated X (Bax)

Bcl-2 and related cytoplasmic proteins are key regulators of apoptosis, including apoptosis inhibitory mechanisms (174). Bcl-2 functions as an oncogene not only by blocking apoptosis, but also by blocking autophagy (175). Bax is a proapoptotic protein which is an essential component of the intrinsic apoptosis signaling pathway, thereby promoting apoptosis (176). Several studies have indicated that some circRNAs upregulated in lung cancer inhibit cell death, and promote disease progression *via* upregulating Bcl-2 and/or downregulating Bax expression. For instance, Wang et al. reported that circVANGL1 overexpression behaved as an oncogene by sponging miR-195, and subsequently activating Bcl-2 to inhibit *in vitro* NSCLC apoptosis (177). Western blot data confirmed that circVANGL1 knockdown increased expression of proapoptotic Bax, while it decreased the expression of antiapoptotic Bcl-2 in LUAD cells (177). Hsa_circ_0109320 inhibited apoptosis in NSCLC by sponging miR-595 to upregulate E2F transcription factor 7 expression, and subsequently upregulating Bcl-2, downregulating Bax and cleaving caspase 3 *in vitro* (178).

Furthermore, several researches also identified the effects of circRNAs on tumorigenesis in *in vivo* studies. For example, Geng et al. observed that hsa_circ_0014130 had oncogenic roles in NSCLC, and functioned as a ceRNA of miR-136-5p, to activate Bcl-2 which inhibited NSCLC apoptosis (179). Qin et al. showed that circPVT1 promoted NSCLC progression by regulating the miR-497/Bcl-2 axis, and inhibiting apoptosis (180). Chen et al. found that circPUM1 was significantly upregulated in both LUAD cell lines and tissue, whereas circPUM1 silencing impaired LUAD proliferation, migration and invasion abilities, and increased apoptosis (181). Mechanistically, circPUM1 could sponge miR-326 to promote expression of its downstream proteins, Bcl-2 and cyclin D1 (181). Furthermore, these studies also indicated that knockdown or silencing hsa_circ_0014130, circPVT1 and circPUM1 inhibited *in vivo* lung cancer tumorigenesis in subcutaneous xenograft mouse models (179–181).

In contrast, circNOL10 was downregulated in lung cancer, and inhibited lung cancer development both *in vivo* and *in vitro* by promoting apoptosis (19). At the molecular level, circNOL10 promoted expression of the transcription factor sex comb on midleg-like 1 (SCML1) by inhibiting transcription factor ubiquitination, and thereby affecting humanin polypeptide family regulation by SCML1. Ultimately, circNOL10 promoted lung cancer cell apoptosis by increasing Bax and caspase-9 expression, and in comparison, decreasing Bcl-2 expression (19). These data indicated that circRNAs regulated lung cancer apoptosis *via* members of the Bcl-2 family, which may be exploited as potential therapeutic targets.

#### The Caspase Family

Caspases are a family of endoproteases, broadly classified by their roles in apoptosis (caspase-3, -6, -7, -8, and -9 in mammals) and inflammation (caspase-1, -4, -5, -12 in humans, and caspase-1, -11, and -12 in mice) (182). Caspase dysregulation underlies several human diseases, including cancer and inflammatory disorders (182). CircRNAs upregulated in lung cancer have played oncogenic roles by regulating caspases expression to inhibit apoptosis. For instance, circRNA ecto-5’-nucleotidase (circNT5E) silencing induced *in vitro* cell apoptosis by increasing the activity of caspase-3 and the cleavages of poly (ADP-ribose) polymerase, *via* miR-134 sponging (183). Moreover, circRNA 100146 functioned as an oncogene in NSCLC cells by interacting with the splicing factor SF3 family (SF3B3, SF3B2 and SF3A1), and binding miR-361-3p and miR-615-5p to regulate multiple downstream mRNAs (i.e., NFAT5, COL1A1, TRAF3 and MEF2C) (184). In further xenograft nude mouse model studies, surgically removed tumors were analyzed and revealed decreased PCNA and p53 levels, and increased caspase-9 and E-cadherin levels in the circRNA 100146 silenced group (184). Yang et al. observed that circRNA TUBA1C accelerated NSCLC progression by sponging miR-143-3p (185). By conducting *in vivo* nude mice studies, these authors also observed that circTUBA1C silencing increased the protein expression of cleaved caspase-3 and Bax (185). These findings indicated that circRNAs are implicated in regulating caspase expression.

### CircRNAs as Potential Biomarkers for Lung Cancer

A biomarker is defined as “a characteristic that is objectively measured and evaluated as an indicator of normal biological processes, pathogenic processes, or pharmacologic responses to a therapeutic intervention” (186). Traditional clinical blood biomarkers of lung cancer include; neuron-specific enolase (NSE), progastrin-releasing peptide (pro-GRP), carcinoembryonic antigen (CEA), CYFRA 21-1, cancer antigen 15 (CA125) and squamous cell carcinoma antigen (SCC). These biomarkers facilitate differential diagnoses, and determine prognostic and monitoring responses to systemic lung cancer therapies. However, these biomarkers still lack comprehensive sensitivity and/or specificity for early diagnoses. Study data from Tarro et al. demonstrated that for early stage I disease detection, NSCLC patients showed a sensitivity of 33.3% for CA19-9, 11.1% for CYFRA 21-1 and CA125, and 0% for CEA; whereas the specificity for all biomarkers was 100% (187). Additionally, Li et al. observed that serum tumor marker sensitivity was 28.46% for CEA, 19.51% for CA 125, 3.25% for NSE, 50.41% for CYFRA 21‐1, 26.02% for SCC and 11.38% for pro‐GRP in lung cancer patients (188).

Therefore in the future, more biomarkers which influence clinical decision-making and improve patient care are required. High numbers of circRNAs have exhibited aberrant expression in human peripheral blood and tissue from lung cancer patients, which are easy to detect. Several studies have demonstrated differential expression patterns of circRNAs under different pathological conditions, thereby indicating the non-invasive biomarker potential of circRNAs in lung cancer (189, 190). These potential circRNA biomarkers are shown (**Table 3**).

**Table 3:** circRNAs as potential biomarker for diagnosis and prognosis in lung cancer.

NO.	Year	circRNA	Level	LC patients/healthy controls	AUC	Sensitivity (%)	Specificity (%)	Sample	Ref
**circRNAs as potential diagnostic biomarkers in lung cancer**									
1	2017	hsa_circ_0013958	up	49/49	0.815	75.5	79.6	tissues	(190)
				30/30	0.794			plasma	
2	2019	hsa_circ_0005962	up	153/54	0.73	71.9	72.22	plasma	(189)
3		hsa_circ_0086414			0.78	77.12	67.67		
4	2020	circ-MEMO1	up	30/25	0.76	56.67	96	plasma	(102)
5	2020	circSATB2	up	59/59	0.66 (non-metastatic)	–	–	plasma	(191)
					0.797(metastatic)				
6	2018	circFARSA	up	55/55	0.71	–	–	plasma	(192)
7	2018	hsa_circ_0102533	up	41/26	0.774 (stage I-II)	–	–	plasma	(193)
					0.728 (stage III-IV)				
8	2020	hsa_circ_0014235	up	30/30	0.8254	–	–	plasma	(194)
9		hsa_circ_0025580			0.80032	–	–		
10	2020	circRNA-002178	up	120/30	0.9956	–	–	plasma	(195)
11	2019	circRNA100146	up	40/40	0.643	72.5	57.5	tissue	(184)
12	2018	circRNA-FOXO3	down	45/45	0.782	80	73.3	tissue	(196)
13	2020	circ-MTHFD2	up	100/100	0.701	90	71	tissue	(197)
14	2020	circ-ACACA	up	60/60	0.7822	–	–	tissue	(100)
15	2018	hsa_circ_ 0000729	up	34/34	0.815	–	–	tissue	(198)
		miR-375	down		0.772	–	–		
16	2018	hsa_circ_0014130	up	46/46	0.878	87	84.8	tissue	(199)
17	2019	hsa_circ_0001073	down	10/10	0.919(LUAD)	–	–	tissue	(200)
18		hsa_circ_0001495	up		0.965(LUSC)	–	–		
19		hsa_circ_0077837	down		0.921	–	–		
20		circPVT1 (hsa_circ_0001821)	up		0.863	–	–		
21	2020	hsa_circRNA_012515	up	83/83	0.89	–	–	tissue	(201)
22	2018	circRNA_102231	up	57/57	0.897	81.2	88.7	tissue	(202)
23	2020	hsa_circ_0007385	up	210/81	0.922	–	–	tissue	(148)
**circRNAs as potential prognostic biomarkers in lung cancer**									
						Independent poor prognostic factor			
24	2019	circ‐TSPAN4 (has_circ_0020732)	up	78/78	0.912(metastatic/ nonmetastatic) 0.907(prognostic)	Not sure		tissue	(203)
25	2020	circHIPK3	up	76/27	–	Yes		tissue	(171)
26	2018	circRNA CDR1as	up	60/20	–	Yes		tissue	(204)
27	2019	circ-PRMT5	up	90/90	–	Yes		tissue	(205)
28	2018	ciRS-7	up	132/132	–	Yes		tissue	(206)
29	2018	circ_0067934	up	159/159	–	Yes		tissue	(207)
	2018			79/79				tissue	(208)
30	2019	circ_0020123	up	55/55	–	Yes		tissue	(209)
33	2018	hsa_circ_0033155	down	40/40	–	Not sure		tissue	(210)
34	2017	circRNA_100876	up	101/101	–	Not sure		tissue	(211)

LC, lung cancer; AUC, area under the cure.

#### Diagnostic circRNAs Biomarkers in Lung Cancer

Several circRNAs of potential diagnostic value have been detected in human plasma, and could be exploited as highly sensitive and specific non-invasive biomarkers for NSCLC. Zhu et al. reported that hsa_circ_0013958 was upregulated in all LUAD tissue, cells and even plasma (190). Additionally, these hsa_circ_0013958 levels were closely related to lymphatic metastasis. The area under the receiver operating characteristic (ROC) cure was 0.815, and sensitivity and specificity for diagnosis were 75.5% and 79.6%, respectively. In addition, plasma hsa_circ_0013958 levels distinguished LUAD cases from healthy controls, with an area under the cure (AUC) of 0.794 (190). These observations indicated that hsa_circ_0013958 could be exploited as a non-invasive biomarker, with high sensitivity and specificity for screening high-risk individuals and early-stage LUAD patients (190). After comparing 153 primary LUAD and 54 normal plasma samples, Liu et al. observed that has_circ_0005962 was upregulated in LUAD, whereas has_circ_0086414 was downregulated (189). The AUC for hsa_circ_0005962 was 0.73, and the optimal cut-off value was 1.21, with a sensitivity of 71.90% and specificity of 72.22%. For hsa_circ_0086414, the AUC was 0.78 and the cut-off value was 0.39, with a sensitivity and specificity of 77.12% and 66.67%, respectively. Additionally, plasma hsa_circ_0005962 showed differential expression patterns in LUAD patients before and after surgical resection, indicating expression may be closely related to patient tumor burden (189). Another study indicated that exosomal circ-MEMO1 levels were higher in NSCLC patient serum, when compared with healthy controls. The AUC was approximately 0.76, with a diagnostic sensitivity and specificity of 56.67% and 96%, respectively (102). Zhang et al. found that circSATB2 was implicated in NSCLC progression by positively regulating fascin homolog 1, actin-bundling protein 1 (FSCN1) expression *via* miR-326 (191). The AUC of exosomal circSATB2 was 0.660 in serum from lung cancer patients, and 0.797 in serum from metastatic lung cancer patients. These observations indicated that exosomal circSATB2 had the potential to provide blood detection indices for the diagnosis of lung and lung cancer metastasis, with high sensitivity and specificity (191). CircFARSA was upregulated in NSCLC patient plasma, and had an AUC diagnostic value of 0.71 (192). Hsa_circ_0102533 served as a blood-based biomarker for stage I-II cancer detection in NSCLC patients (AUC = 0.774), and stage III-IV detection in NSCLC patients (AUC = 0.728) (193). Another study revealed diagnostic indices for hsa_circ_0014235 (AUC = 0.8254) and hsa_circ_0025580 (AUC = 0.80032) in LUSC plasma samples, suggesting high diagnostic values for LUSC (194). Wang et al. compared serum samples from 30 healthy controls and 120 LUAD patients without treatment, and observed that circRNA-002178 was upregulated, with an AUC of 0.9956 (195).

In addition, circRNA expression indices, with potential diagnostic value, have been detected in cancer tissue only, but not plasma. Chen et al. demonstrated that the AUC for circRNA100146 was 0.643 in NSCLC tissue, and the sensitivity and specificity were 72.5% and 57.5%, respectively (184). Zhang et al. observed that the AUC for circRNA-FOXO3 was 0.782, and sensitivity and specificity indices were 80.0% and 73.3%, respectively (196). Geng et al. also reported that the AUC of circ-MTHFD2 was 0.701, with a cut-off value of 3.534, and 90% sensitivity and 71% specificity (197). The diagnostic accuracy of circ-ACACA was assessed using ROC curve analysis, and showed that the AUC was 0.7822 (100). Li et al. observed that hsa_circ_ 0000729 was upregulated in LUAD tissue and sponged miR‐375, both of which had significant diagnostic accuracy, with AUC values of 0.815 and 0.772, respectively (198). Hsa_circ_0014130 exhibited significant overexpression in NSCLC tissue, with an AUC of 0.878 and an optimal cutoff value 0.573, with sensitivity and specificity indices measured at 87.0% and 84.8%, respectively (199). Wang et al. in an RNA-Seq analysis observed that four circRNAs from 17,952 circRNAs, had diagnostic values for NSCLC (200). Specifically, the AUC for hsa_circ_0001073 was 0.919 in LUAD tissue, and 0.965 for hsa_circ_0001495 in LUSC tissue, indicating both molecules could serve as effective diagnostic biomarkers for LUAD and LUSC prediction, respectively. For the other circRNAs, the AUC for hsa_circ_0077837 was 0.921, and for hsa_circ_0001821 (circPVT1), 0.863. However, these circRNAs were unable to distinguish LUAD and LUSC subtypes (200).

Furthermore, several circRNAs have exhibited high diagnostic accuracy, while the aberrant expression of these circRNAs has been closely associated with cancer prognoses. Fu et al. observed that hsa_circRNA_012515 exhibited a high diagnostic accuracy for NSCLC, with an AUC of 0.89 (201). Kaplan-Meier (K-M) survival analyses also indicated that OS and disease free survival (DFS) indices were considerably shortened in patients expressing high levels of hsa_circRNA_012515, when compared to those patients with low expression (201). Zong et al. indicated that circRNA_102231 expression was significantly upregulated in LUAC tissue, and was associated with advanced TNM stage, lymph node metastasis and poor OS in lung cancer patients, with an AUC of 0.897 (202). CircRNA_102231 also exhibited a good sensitivity of 81.2%, and specificity of 88.7%. Hsa_circ_0007385 was upregulated in NSCLC tissue with an AUC of 0.922; multivariate regression analyses indicated that the high expression of this circRNA independently predicted a worse OS status (148). These studies showed that some circRNAs may be considered good (non-invasive) biomarkers for lung cancer diagnostics, and some are useful as prognostic biomarkers.

#### Prognostic circRNA Biomarkers in Lung Cancer

CircRNAs exhibited different expression patterns and levels in lung cancer tissue. These expression levels are closely related to the stage and prognosis of disease, potentially making them new prognostic biomarkers in clinical settings. For instance, Ying et al. observed that circ‐TSPAN4 (has_circ_0020732) expression levels were increased in LUAD tissue and cell lines, suggesting it as a promising prognostic biomarker for LUAD patients (203). The AUC for circ‐TSPAN4 expression levels for metastatic versus non-metastatic LUAD patients was 0.912. More importantly, the OS status for patients with high circ‐TSPAN4 expression levels was significantly shorter than patients with low levels, and the prognostic utility of circ‐TSPAN4 for LUAD patients was high, with an AUC of 0.907 (203). Moreover, Chen et al. observed that circHIPK3 was upregulated in NSCLC, while its linear counterpart, linear HIPK3 (linHIPK3) was significantly downregulated in diseased tissue, when compared to adjacent normal tissue (171). The expression ratio between circHIPK3 and linHIPK3 was significantly higher in tumors when compared to normal tissue, and indicated poor survival, especially for advanced-stage NSCLC patients.

In addition, several circRNAs showed higher expression in NSCLC samples than the normal counterparts that was highly associated with TNM stage and lymph node metastasis, but not associated with other factors such as, gender, age, smoking, histology, etc, which could function as an independent poor prognostic factor. For example, Zhang et al. showed that circRNA CDR1as was upregulated in NSCLC tissue (204). Further data indicated that patient’s expressing high circRNA CDR1as levels had shorter OS than those with low levels. Similar results were also generated for circRNA PRMT5 (circ-PRMT5); high expression levels indicated lung cancer patients were more likely to develop poor progression-free survival (PFS) and OS (205). In their investigation, Yan et al. indicated that high ciRS-7 expression was correlated with shorter DFS and OS status, and multivariate Cox’s proportional hazard regression analyses showed this high expression was an independent factor for predicting a poor prognosis (206). Both Wang et al. and Zou et al. reported that circ_0067934 expression was significantly increased in NSCLC tissue and cell lines (207, 208). K-M curves showed that high expression of this circRNA was an independent risk factor for OS status in patients with NSCLC in both studies. The K-M analyses by Wan et al. indicated that high circ_0020123 expression levels were associated with decreased OS in NSCLC patients (209).

Furthermore, several studies have demonstrated the biomarker potential of other circRNAs, however their role as independent prognostic factors requires further study. For instance, Gu et al. observed that the aberrant expression of hsa_circ_0033155 in NSCLC tissue was correlated with lymphatic metastasis, however no other significant indices were associated with other clinicopathological characteristics (210). Yao et al. proposed a close correlation between circRNA_100876 upregulated expression and lymph node metastasis and tumor staging (211). Moreover, K-M survival analyses demonstrated that the OS for NSCLC patients with high circRNA_100876 expression levels was significantly shorter for patients with low levels (211). Thus, these findings suggested that circRNAs could serve as prognostic markers for lung cancer, however they require further investigation to prove their utility as independent prognostic factors.

### The Therapeutic Potential of circRNAs in Lung Cancer

#### The Role of circRNAs in Lung Cancer Drug Resistance

The greatest hurdle to targeted cancer therapy is the inevitable emergence of drug resistance (212); tumors can develop this resistance during the early or late phase of drug treatment. Resistance is typically classified into two therapeutic categories: (1) intrinsic or primary resistance and, (2) acquired or secondary resistance (212). In recent years, an increased volume of studies have focused on mechanisms of drug resistance in lung cancer (213, 214). Such mechanisms include; the Thr790Met mutation in the epidermal growth factor receptor (EGFR) which induces resistance to tyrosine kinase inhibitors (TKIs) (215), and tumor-reprogramming of the lung microenvironment which induces resistance to angiogenesis and immune checkpoint molecular target therapies in lung cancer (216).

However, in recent years, the role of circRNAs in drug resistance has gained considerable traction with different research groups. Here, we summarize the current data exemplifying the role of circRNAs in therapeutic resistance and related mechanisms of disease.

##### Platinum Cytotoxic Drugs: Cisplatin (CDDP)

Cisplatin mainly exerts its cytotoxic effects in tumor cells *via* the generation of DNA-platinum adducts, and subsequent DNA damage responses (212). Despite major clinical successes over recent decades, cisplatin-mediated drug resistance in tumor cells has hindered the clinical utility of this drug (217). Notwithstanding this, a number of mechanisms have emerged contributing to resistance onset. For instance, STAT3 may be attributable to CDDP resistance, and its inhibitors could reverse CDDP-resistance (218). Recently, circRNAs upregulated in NSCLC were found to promote CDDP resistance by activating STAT3 expression; Dong et al.reported that circ_0076305 positively regulated CDDP resistance by upregulating STAT3 by targeting miR-296-5p (219). Moreover, Xu et al. found that circAKT3 inhibited glycolysis and CDDP sensitivity in lung cancer cells by regulating the miR-516b-5p/STAT3 axis (220).

In addition, several other circRNAs were overexpressed in NSCLC, and positively regulated CDDP resistance *via* other mechanisms. Hong et al. observed that circ-CPA4 promoted cell invasion and EMT processes in NSCLC *via* the let-7 miRNA/PD-1 axis (221). In addition, NSCLC cell derived PD-L1 exosomes self-regulated cell stemness to increase NSCLC resistance to CDDP, blockading PD-L1 sensitized chemoresistant NSCLC cells to CDDP (221). CircRNACDR1as promoted LUSC metastasis by sponging miR-671-5p and regulating Golgi trafficking (222), thus, participating in CDDP-resistance in NSCLC. Moreover, Zhao et al. indicated that circRNACDR1as regulated stemness properties mediated by CDDP resistance in NSCLC cells by targeting the miR−641/HOXA9 axis (223). Huang et al. screened 31 × eukaryotic initiation factor 3 (EIF3)-derived circRNAs, and correlated two circEIF3 molecules (hsa_circ_0004350 and hsa_circ_0092857) with CDDP drug sensitivity in lung cancer (224); downregulation of these molecules reversed CDDP resistance in lung cancer cells. Pang et al. observed that levels of circ‐PRMT5 and EV3‐like DNA‐directed polymerase ζ catalytic subunit (REV3L) were markedly increased, while miR‐4458 was downregulated in CDDP-resistant NSCLC tissue and cells (225). Circ‐PRMT5 absence contributed to CDDP sensitivity *via* the miR‐4458/REV3L axis. Importantly, circ‐PRMT5 silencing affected CDDP treatment to expedite an *in vivo* decrease in tumor growth (225). Li et al. observed that circ_0072083 depletion contributed to CDDP-triggered inhibition of NSCLC tumors *via* the miR-545-3p/CBLL1 axis (226). Lu et al. proposed that hsa_circ_0096157 expression contributed to CDDP resistance in NSCLC cells by regulating the cell cycle signaling pathway, EMT processes and the expression of apoptosis associated proteins (227). Hsa_circ_0085131 enhanced CDDP-resistance in NSCLC cells by sponging miR-654-5p to upregulate ATG7, leading to cell autophagy (228). Circ_0000376 functioned with oncogenic roles in NSCLC, and enhanced *in vitro* NSCLC CDDP-resistance by repressing miR-384 (229). Xiao et al. reported that circRNA_103762 expression was upregulated in CDDP-resistant NSCLC cells, and enhanced multidrug resistance by inhibiting CHOP (DNA damage inducible transcript 3) expression in NSCLC cells (230).

However, several circRNAs downregulated in lung cancer have different roles in CDDP-resistance in lung cancer, distinct to the aforementioned circRNAs. Huang et al. indicated that hsa_circ_0001946 exhibited tumor suppressive roles in lung cancer cells, and affected NSCLC cell sensitivity to CDDP *via* modulation of the Nucleotide excision repair signaling pathway (231). Additionally, a recent study showed that circRNA epithelial splicing regulatory protein-1 (ESRP1) was significantly downregulated in chemoresistant lung cancer cells (232). CircRNA cESRP1 was sensitized to small cell lung cancer (SCLC) cells upon chemotherapy (i.e., doxorubicin, CDDP and etoposide) by sponging miR-93-5p, and upregulating expression of the miR-93-5p downstream target, Smad7/p21(CDKN1A). This formed a negative feedback loop that regulated transforming growth factor-β (TGF-β) mediated EMT processes (232). These studies highlighted the crucial role of circRNAs in CDDP resistance, in both NSCLC and SCLC, and as such may be considered potential novel therapeutic targets for drug resistance in lung cancer.

##### Antimetabolite Drugs: Pemetrexed and Gemcitabine

Pemetrexed is a novel multitargeted antifolate that inhibits ≥ three enzymes involved in folate metabolism and purine and pyrimidine synthesis (233). Pemetrexed and CDDP combination chemotherapies are widely used to treat NSCLC (234). Mao et al. observed that circRNA CDR1-as was highly expressed in pemetrexed and CDDP resistant LUAD tissues and cell lines (235). CircRNA CDR1-as promoted pemetrexed and CDDP chemoresistance *via* the EGFR/PI3K signaling pathway in LUAD (235). Zheng et al. reported that circPVT1 overexpression was positively related to chemotherapy insensitivity in LUAD patients (236). Mechanistically, circPVT1 was shown to contribute to pemetrexed and CDDP chemotherapy resistance *via* the miR-145-5p/ABCC1 axis (236).

Gemcitabine (2’,2’-difluoro 2’-deoxycytidine, dFdC) is the most important cytidine analogue developed since cytosine arabinoside (Ara-C) (237). The cytotoxic activity of gemcitabine may result from several actions against DNA synthesis (237). The combination of gemcitabine and CDDP chemotherapy may be a viable treatment for advanced NSCLC (238). Lu et al. observed that circPVT1 expression was decreased after gemcitabine and CDDP combination treatment; circPVT1 expression in chemotherapy-resistant patients was higher than chemotherapy-sensitive patients (239). Thus, it may be feasible to determine therapy effects post-chemotherapy by detecting circPTV1 expression in patient serum. However, Tong et al. observed that circ-SMARCA5 overexpression enhanced chemosensitivity to gemcitabine and CDDP in treated cells, when compared to overexpression control cells (240). These findings indicated that circRNAs may facilitate rescue for pemetrexed/gemcitabine and CDDP combined chemotherapy resistance in patients.

##### Plant Derived Chemotherapy Drugs: Paclitaxel (taxol)

Paclitaxel (taxol) was the first member of the taxane family used for cancer chemotherapy; taxanes exert their cytotoxic effects by arresting mitosis *via* microtubule stabilization, resulting in cellular apoptosis (241). Paclitaxel use has become a broadly accepted option for the treatment of patients with NSCLC (241). A recent study observed that circ_0011292 facilitated tumorigenesis and paclitaxel resistance in NSCLC by regulating the miR-379-5p/TRIM65 axis, suggesting circ_0011292 was a promising therapeutic target for NSCLC chemotherapy (242). Circ_0011292 silencing also reduced paclitaxel resistance *in vivo (* 242). However, Li et al. indicated that circ_0002483 overexpression significantly inhibited NSCLC cell proliferation and invasion *in vitro* and *in vivo*, and enhanced NSCLC sensitivity to taxol by sponging miR-182-5p to release the inhibition on GRB2, FOXO1 and FOXO3 mRNAs (243). These studies demonstrated the different roles circRNA exert towards paclitaxel resistance in lung cancer, suggesting their potential as therapeutic strategies in the future.

##### Targeted Drugs: Epidermal Growth Factor Receptor Tyrosine Kinase Inhibitors (EGFR-TKIs)

These molecules, which include gefitinib, erlotinib and osimertinib (AZD9291), have become important treatment options for NSCLC patients with EGFR sensitive mutations (244). The acquired resistance mechanisms are currently unclear, except for the Thr790Met mutation (215). Recent studies have indicated that circRNAs may play important roles in EGFR-TKI resistance. For instance, Zhou et al. reported that hsa_circ_0004015 contributed to disease progression and gefitinib resistance in NSCLC patients; mechanistically, circ_0004015 sponged miR-1183 and subsequently targeted 3-phosphoinositide dependent protein kinase 1 (PDPK1) (245). PDPK1 is a classic effector of the EGF signaling pathway, which prevents apoptosis and mediates drug resistance in pancreatic cancer (246). Wen et al. found that hsa_circ_0000567 was upregulated and hsa_circ_0006867 downregulated in gefitinib-resistant NSCLC cell lines, when compared to sensitive cells, indicating these circRNAs may be implicated in acquired gefitinib resistance *via* a circRNA-miRNA-mRNA interactive network (247). Moreover, by analyzing 52 NSCLC patients treated with gefitinib, Liu et al. reported that elevated hsa_circ_0109320, identified from 1,377 circRNAs, was associated with longer PFS in gefitinib-treated NSCLC patients (248). Thus, this circRNA may be a potential biomarker for EGFR-TKI efficacy in these patients.

In addition, Joseph et al. observed that hepatocyte growth factor/c-Met regulated expression of the SUMO-activating enzyme subunit 2(SAE2) and circRNA CCDC66 to increase gefitinib and erlotinib resistance in LUAD cells (249). Mechanistically, SAE2 maintained protein stability, including AAA domain-containing 3A and the EMT markers, vimentin and paxillin, which were crucial for metastatic potential and drug resistance. Critically, these bio-parameters correlated with prognoses in LUAD patients (249).

Furthermore, Ma et al. observed that hsa_circRNA_0002130 was highly expressed in osimertinib-resistant NSCLC cells, and in serum exosomes derived from osimertinib-resistant NSCLC patients (250). Mechanistically, this circRNA facilitated osimertinib-resistance in NSCLC patients by sponging miR-498 to upregulate the GLUT1, hexokinase 2 and LDHA (250). Chen et al. identified 15,504 circRNAs that were differentially expressed in AZD9291-resistant NSCLC cell lines versus control cell lines, and revealed that circRNAs may have roles in NSCLC-AZD9291 resistance. These authors also suggested these circRNAs may be promising molecular candidates for gene therapy (251). These findings demonstrated the importance of circRNAs in TKI-resistance, and their potential as biomarkers for TKI treatment efficacy. However, further studies are required to explore circRNA molecular mechanisms so that target gene therapy studies may proceed to clinical trials.

##### Immunotherapeutic Drugs: Programmed Cell Death 1/Programmed Cell Death-Ligand 1 (PD-1/PDL-1)

Immune checkpoint inhibitors (ICIs) have dramatically changed the landscape of NSCLC treatment. The PD-1/PDL-1 inhibitors now forms part of first-line NSCLC mono-therapy treatments, or combined with chemotherapy or chemoradiotherapy in patients with stage III unresectable NSCLC (252). Anti-PD-1/PDL-1 ICIs have indicated promising efficacies (~30% response rates), and improved survival of patients with metastatic NSCLC (253). The mechanism of drug resistance during immunotherapy remains unclear, however circRNAs have been shown to exert important functions in NSCLC immunotherapy resistance. CircRNA fibroblast growth factor receptor 1 (circFGFR1/hsa_circ_0084003) promoted NSCLC progression and resistance to anti-PD-1-based therapy (17). CircFGFR1 directly interacted with miR-381-3p, then upregulated the expression of its target gene, C-X-C motif chemokine receptor 4 (CXCR4). Additionally, Luo et al. observed that lung cancer patients with positive PDL-1 expression (≥ 1%) expressed higher level of hsa_circ_0000190 (254). Moreover, long-term follow-up of immunotherapy treated cases indicated that upregulated plasma hsa_circ_0000190 levels correlated with poor responses to systemic therapy and immunotherapy (254). These findings demonstrated that circRNAs played key roles in ICI-resistance, providing potential therapeutic targets for these mechanisms. Similarly, these data also indicated that circRNAs are potential biomarkers for ICI treatment efficacy.

#### The Role of circRNAs in Radiotherapy Resistance of Lung Cancer

Radiotherapy can be applied in local and regional advanced NSCLC with no surgical chance, as neoadjuvant in the group that has the potential to have surgery and can be applied as adjuvant considering some risk factors after surgery (255). Similarly, radioresistance mechanisms in NSCLC are currently unclear. One potential radioresistance mechanism involves the generation of exosomes. Exosomes are 40–150 nm vesicles released by cancer cells and contain pathogenic components, such as proteins, mRNAs, DNA fragments, non-coding RNAs and lipids. It has been proposed that radiation-derived exosomes may promote radioresistance (256). For instance, Fan et al. observed that both intracellular and extracellular miR-1246 levels were upregulated after irradiation in a time-dependent pattern, resulting in increased NSCLC radioresistance *via* mTOR-inhibited autophagy activation (257). Moreover, circRNA CDR1as sequestered miR-1246 and antagonized its effects towards radioresistance mechanisms in NSCLC cell lines (257). Accordingly, it suggested that circRNAs could play vital roles in radioresistance mechanisms in lung cancer, however more studies are required to explore such mechanisms initiated by circRNAs in radioresistance.

## Conclusion and Perspectives

RNA-seq technologies have provided unprecedented insights into the human genome; the clinical value of circRNAs has been unraveled for various diseases, including lung cancer. As outlined here, circRNAs participate in lung cancer proliferation, migration, invasion and apoptosis, and have the extraordinary potential to be recognized as biomarkers for molecular therapeutics. As good biomarkers, circRNAs have stable molecular structure and are more abundant than liner RNAs. However, research in this area is far from complete, therefore we propose three critical research areas which will define the success of these molecules as biomarkers; (1) The role of circRNAs in other lung cancer pathological processes is little known, therefore more studies are required to investigate tumor microenvironments, molecular heterogeneity and inflammation, etc. (2) More circRNA biomarker research must be conducted to distinguish different pathological types of lung cancer. (3) More comprehensive and in-depth clinical trials must be conducted to verify these biomarkers as potential therapeutics for lung cancer. (4) In-depth studies on cells, animals and population cohorts are needed to further explore the value of circRNAs in evaluating the prognosis of lung cancer.

By implementing these three key strategies, we will gain a better understanding of the regulatory roles of circRNAs in lung cancer, as they will provide new molecular insights into circRNA mechanisms behind the disease. Thus, this review has systematically and comprehensively highlighted the recent advances in circRNAs in lung cancer etiology, and has exemplified the diagnostic and therapeutic potential of circRNAs in this disease.

## Author Contributions

H-HC and T-NZ contributed equally to this work. H-HC and T-NZ wrote the manuscript. Q-JW and Y-HZ is responsible for research supervision. Y-HZ is responsible for funding acquisition. All authors contributed to the article and approved the submitted version.

## Funding

This work was supported by the National Key R&D Program of China (No. 2017YFC0907401 to Y-HZ), the Natural Science Foundation of China (No. 82073647 to Q-JW), and LiaoNing Revitalization Talents Program (No. XLYC1907102 to Q-JW and No. XLYC1802095 to Y-HZ).

## Conflict of Interest

The authors declare that the research was conducted in the absence of any commercial or financial relationships that could be construed as a potential conflict of interest.
